# Prediction of genetic contributions to complex traits using whole genome sequencing data

**DOI:** 10.1186/1753-6561-8-S1-S68

**Published:** 2014-06-17

**Authors:** Chen Yao, Ning Leng, Kent A Weigel, Kristine E Lee, Corinne D Engelman, Kristin J Meyers

**Affiliations:** 1 Department of Dairy Science, University of Wisconsin, 1675 Observatory Drive, Madison, WI 53706, USA; 2 Department of Statistics, University of Wisconsin, 1220 Medical Sciences Center, 1300 University Ave, Madison, WI 53706, USA; 3 Department of Ophthalmology and Visual Sciences, University of Wisconsin Medical School, 1069 WARF Building, 610 North Walnut Street, Madison, WI 53726, USA; 4 Department of Population Health Sciences, University of Wisconsin School of Medicine and Public Health, Madison, WI53726, USA

## Abstract

Although markers identified by genome-wide association studies have individually strong statistical significance, their performance in prediction remains limited. Our goal was to use animal breeding genomic prediction models to predict additive genetic contributions for systolic blood pressure (SBP) using whole genome sequencing data with different validation designs.

The additive genetic contributions of SBP were estimated via linear mixed model. Rare variants (MAF<0.05) were collapsed through the k-means method to create a "collapsed single-nucleotide polymorphisms." Prediction of the additive genomic contributions of SBP was conducted using genomic Best Linear Unbiased Predictor (GBLUP) and BayesC*π*. Estimates of predictive accuracy were compared using common single-nucleotide polymorphisms (SNPs) versus common and collapsed SNPs, and for prediction within and across families.

The additive genetic variance of SBP contributed to 18% of the phenotypic variance (*h^2 ^*= 0.18). BayesC*π *had slightly better prediction accuracies than GBLUP. In both models, within-family predictions had higher accuracies both in the training and testing set than didacross-family design. Collapsing rare variants via the k-means method and adding to the common SNPs did not improve prediction accuracies. The prediction model, including both pedigree and genomic information, achieved a slightly higher accuracy than using either source of information alone.

Prediction of genetic contributions to complex traits is feasible using whole genome sequencing and statistical methods borrowed from animal breeding. The relatedness of individuals between the training and testing set strongly affected the performance of prediction models. Methods for inclusion of rare variants in these models need more development.

## Background

The genetic architecture underlying complex traits is hypothesized to involve numerous individual loci, of varying frequency, each with small to moderate effects.Genome-wide association studies (GWAS) have generally focused on single nucleotide polymorphisms (SNPs) occurring at a minor allele frequency (MAF) >0.05 with strict statistical criteria for inclusion in the predictive models (eg, individual SNPs with *p*value <5 × 10^−8^).To date, loci from GWAS for quantitative traits such as blood pressure and height have provided only limited ability to explain the variability of complex traits, resulting in "missing heritability" [1], and their usage for disease prediction has been limited [2].

An alternative approach for explaining the heritability and improving prediction of the additive genetic contributions (known as "breeding value" in animal breeding) to complex traits is the use of whole genome markers jointly [3,4]. As reviewed by de los Campos et al, whole genome prediction methods, borrowed from animal breeding, provide the potential to greatly improve the prediction of genetic risk for complex traits in humans, as compared to prediction using only specific susceptibility loci from GWAS [2]. Further improvement in prediction models might come from the inclusion of rare variants. Through whole genome sequencing, there is an unprecedented opportunity for predicting the individual additive genetic contributions for complex traits through the inclusion of variants across the frequency and effect size spectrums.

In this study, we applied animal breeding whole genome prediction methods to data provided by the Genetic Analysis Workshop 18 (GAW18) to predict the additive genetic contributions of a complex trait, systolic blood pressure (SBP), in humans. As part of this study, we explored 2 methods for validation and the k-means method to collapse and include rare variants into the prediction model.

## Methods

### Phenotypic values

We used the real data provided by GAW18, including information from up to 4 measurements of SBP per individual. Observed variation in SBP is a function of genetic and environmental factors [5]. A linear mixed model was applied to partition variance of SBP after accounting for 5 fixed effects (**β **and *p *= 5).Variance is partitioned into the additive genetic effect (**u**) and the repeated environmental effect (**c**) of each individual. The additive genetic effect (**u**) was estimated based on degree of additive relatedness determined from pedigree structure. The repeated environmental effect was the environmental effect on an individual's phenotype that is constant across (or common to) repeated measures on that individual, and independent between different individuals, which was defined by fitting individual identity as an additional random effect [6].The linear mixed model below was applied to 2189 records (*n*) of 916 individuals (*q*) without missing phenotype, and included information from every examination,

(1) y=Xβ+Zu+Tc+e

where **y **is an *n * × 1 vector of SBP measurements; **X **is an *n × p *matrix containing fixed effects variables including year of examination, age, sex, medications usage, and tobacco smoking; **β **is a *p * × 1 vector of fixed effects parameters;**Z **is an *n* × *q *matrix containing dummy variables and relating each of the additive genetic effect to an individual's phenotype; $u\sim N\left(0,A\sigma_{u}^{2}\right)$ is a *q * × 1 vector of additive genetic effects for all individuals where **A **is a *q * × *q *pedigree-based kinship matrix; T is an *n * × *q *matrix containing dummy variables and relating each of the repeated environmental effect to an individual's phenotype; $c\sim N\left(0,I\sigma_{c}^{2}\right)$ is a *q * × 1 vector of random repeated environmental effects where **I **is a *q * × *q *identity matrix assuming independent repeated environmental effects among different individuals; and error term $\text{e}\sim N\left(0,\text{I}\sigma_{e}^{2}\right)$. The mixed model equation was solved with the restricted maximum likelihood method using the "pedigreemm" R-package version 0.2-4. The estimated additive genetic contributions were taken as the estimated random additive genetic effects $\hat{\text{u}}$, and were used as the independent variable in the genomic prediction models.

The narrow-sense heritability [5] (hereafter "heritability") was calculated from the variance components estimated in model (1) as shown in model (2):

(2) $${\hat{h}}^{2}=\frac{{\hat{\sigma }}_{u}^{2}}{{\hat{\sigma }}_{u}^{2}+{\hat{\sigma }}_{c}^{2}+{\hat{\sigma }}_{e}^{2}}$$

### Whole genome sequencing data

Based on 139 unrelated founders from all 20 families, the whole genome sequence markers provided in GAW18 were pruned using PLINK 1.07 [7], keeping markers with linkage disequilibrium coefficient *r^2^*<0.9. The whole genome prediction models were applied to 835 individuals from 20 families with both genotype and phenotype data. Common SNPs with MAF ≥0.05 were coded to an additive genetic model (0, 1, or 2 minor alleles) using the rounded dosages given by GAW18.

Even though many approaches have been developed for collapsing rare variants to test in association studies [8], approaches for including rare variants in prediction models have not been explored. In this study, a "collapsed SNP" was generated from a vector of 100 rare SNPs based on physical position within the chromosome with k-means method [9], which is a popular clustering method in the fields of statistical learning and pattern recognition [10].

To be consistent with the 3 level genotypes of common SNPs, the k-means method was applied to each 100-rare-SNP vector generated to partition genotypes into 3 clusters, in which each of 835 individuals belonged to the cluster with the nearest mean of individuals in that cluster to minimize the within-cluster sum of square error. After clustering, individuals in the same cluster are expected to be genetically closer to each other compared to individuals from different clusters. Individuals in the cluster with the largest, medium, and smallest cluster size were assigned a collapsed SNP genotype to be 0, 1, and 2, respectively, and the MAF was calculated. A total of 26,845 collapsed SNPs were formed from 2,683,921 rare SNPs.

**Figure 1:**
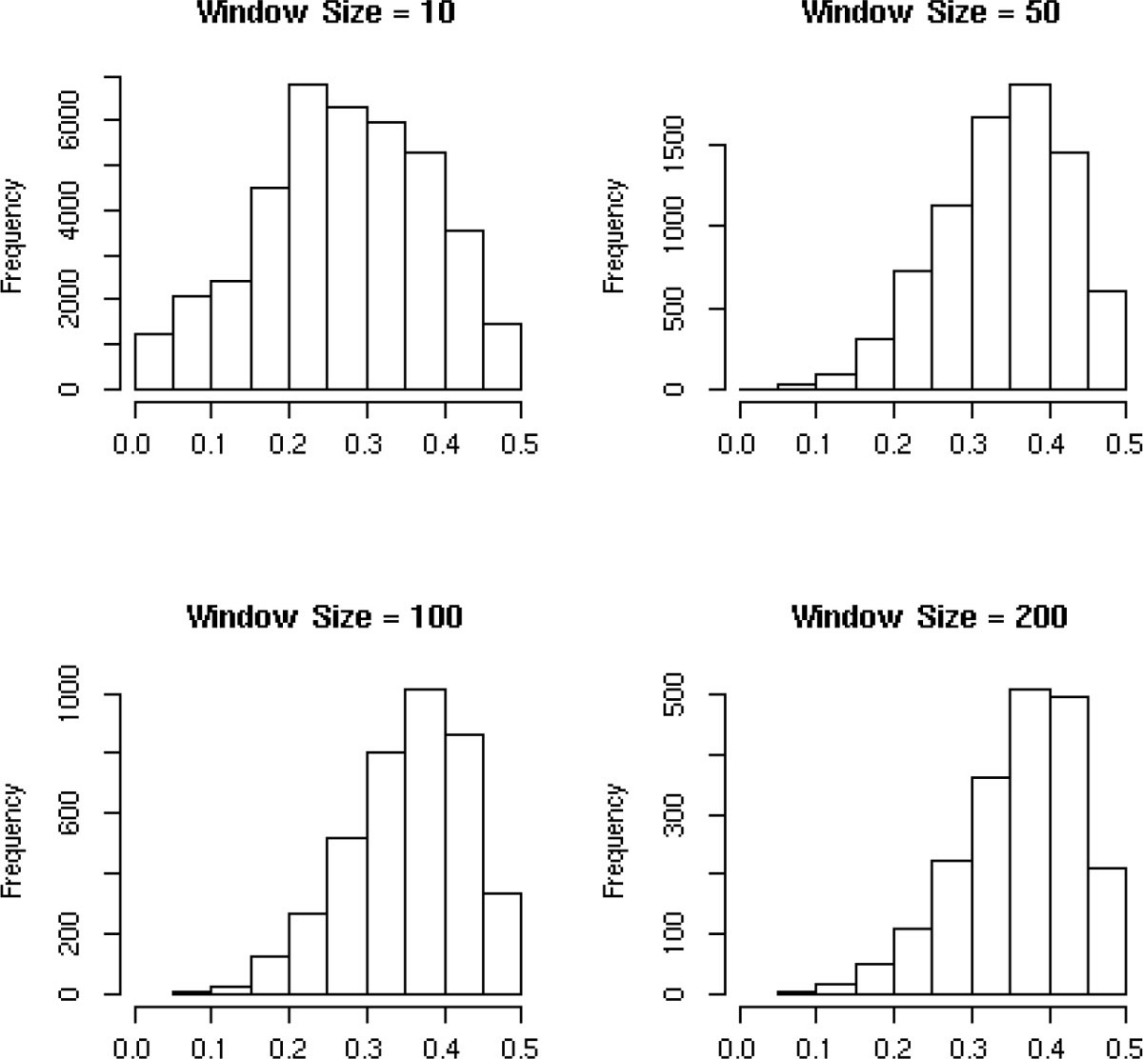
**The minor allele frequency (x-axis distribution of collapsed SNPs using the k-means method and different window sizes**.

By testing different window sizes across all chromosomes, we found that the number of collapsed SNPs with a MAF<0.05 decreased as the window size increased (Figure 1). A larger window size, however, will provide less information after collapsing. The window size, 100, was chosen to minimize the number of collapsed SNPs with a MAF<0.05, and keep information after collapsing maximally. When applying the prediction models, 2sets of SNPs were considered: "set 1" with 964,208 common SNPs and "set 2" with 991,053 SNPs, including both common and collapsed SNPs formed by rare variants.

### Whole genome prediction models

The whole genome prediction models, genomic Best Linear Unbiased Predictor (GBLUP) and BayesC*π*, were trained using 2validation designs described in the next section. The general statistical model is below, and the predicted genomic value of each individual was defined as the fitted value ŷ′:

(3) y′=μ+Ma+e′

where y′ is the estimated genetic value $\hat{\text{u}}$ from model (1), **μ **is the overall mean, **a **is a vector of random additive effects of all loci (of SNP set 1 or set 2) with genotype matrix **M**, and error term $\text{e}\prime \sim N\left(0,\text{I}\sigma_{e\prime }^{2}\right)$.

In GBLUP, it is assumed that $\text{a}\sim N\left(0,\text{K}\sigma_{a}^{2}\right)$, that is, the same variance is shared by all loci, where **K **is the whole genome marker-based relationship matrix. The estimates of marker effect $\hat{\text{a}}$ are obtained following the solution of mixed-model equations in Meuwissen et al[11].

In BayesC*π*, besides sharing the common variance among all loci, a prior distribution was assigned to the additive effect of each locus depending on the variance $\sigma_{a}^{2}$ and the probability *π *that the given SNP has zero effect (formula (4)). The algorithm was implemented as in Habier et al[12].

(4) $$a|\pi ,\sigma_{a}^{2}=\{\begin{array}{cc} 0 & \text{with}\;\text{probability}\;\pi ,\\ N\left(0,\sigma_{a}^{2}\right) & \text{with}\;\text{probability}\;\left(1-\pi \right). \end{array}$$

Both GBLUP and BayesC*π *were implemented via Gibbs sampling. The ratio $\sigma_{a}^{2}/\sigma_{e}^{2}$ in GBLUP was set to 2/3, according to the output of BayesC*π*. The initial value for *π *in BayesC*π *was set as 0.9. The total number of iterations in both models was 12,000 with a burn-in of 2000 and a mining rate of 10. Longer chains (total of 40,000 with a burn-in of 5000 and a mining rate of 10) did not improve the correlation between predicted value and true value. The correlations were also consistent among multiple short chains with the same length of 12,000 iterations.

### Validation design for prediction models

Two different validation designs were used to evaluate the predictive ability of different models and using different SNP sets. The first was a within-family prediction with the first 3generations from all 20 families (528 individuals) in the training set (TRN), and their descendants from the fourthand fifthgenerations (307 individuals) in the testing set (TST). The second was an across-family prediction using 5-fold cross-validation. To balance sizes of training and TSTs in each replicate of cross-validation, 20 families were ranked by their family sizes, and then every five families were randomly assigned to five different folds (four families in each fold) to get about 668 individuals in TRN and about 167 in TST.

### Predictive accuracy

The accuracies of genomic prediction were measured by the correlation of the estimated additive genetic contributions $\left(y\prime =\hat{u}\right)$ with their genomic prediction values (y′) from model (3).

The prediction accuracies were compared between genome and pedigree based additive genetic contributions $\left({\hat{u}}_{p}\right)$ for individuals in within-family prediction TST with at least 1 parent in TRN. The ${\hat{u}}_{p}$ was calculated with formula (5).

(5) $${\hat{u}}_{p}=\;\{\begin{array}{cc} {\hat{u}}_{F}, & \text{if}\;\text{only}\;\text{father}\;\text{in}\;\text{TRN}.\\ {\hat{u}}_{M}, & \text{if}\;\text{only}\;\text{mother}\;\text{in}\;\text{TRN}.\\ 0.5\times {\hat{u}}_{F}+0.5\times {\hat{u}}_{M}, & \text{o}.\text{w}. \end{array}$$

where ${\hat{u}}_{F}$ and ${\hat{u}}_{M}$ are estimated additive genetic contributions of the father and mother of the individual. The linear models in (6) were then fitted, and the R^2 ^values (coefficient of determination of the linear regression) from the model fitting were reported to be the predictive accuracies using genomic only, parent average only, and both genomic and parent average information.

(6) $$\begin{array}{cc} \hat{\text{u}}=b_{1}\hat{\text{y}}\prime +e_{1}\\ \hat{\text{u}}=b_{2}{\hat{\text{u}}}_{p}+e_{2} & \\ \hat{\text{u}}=b_{3}\hat{\text{y}}\prime +b_{4}{\hat{\text{u}}}_{p}+e_{3} & \end{array}$$

## Results and discussion

The additive genetic contributions of SBP ranged from −18.9 to 15.8 with mean 0.2 and SD 3.5. The estimated variance components, additive genetic variance $\left(\sigma_{g}^{2}\right)$, repeated environmental variance $\left(\sigma_{c}^{2}\right)$, and error variance $\left(\sigma_{e}^{2}\right)$, of model (1) were 44.4, 61.5, and 135.0, respectively. The estimated heritability of SBP was calculated to be 0.18 using formula (2), which means that 18% of phenotypic variance was a result of additive genetic contributions. The reported heritability estimates of SBP in previous studies ranged from 0.24 to 0.37 [13-15]. The slightly lower heritability estimates from this study could result from different methods for estimation or different populations and environments [16]. When the data contained repeated measurements, failure to model a repeated environmental effect would inflate estimates of heritability by interpreting the covariance because of repeated environmental effects as covariance among a series of clones with a coefficient of coancestry of 0.5 [6]. Our linear mixed model (model 1) incorporated repeated environmental measures, therefore minimizing this possibility.

Table 1 outlines correlations between additive genetic contributions of SBP and predicted genomic values and corresponding mean square errors (MSE) in within-family validation and across-family prediction with GBLUP and BayesC*π*. In general, the BayesC*π *outperformed GBLUP based on both correlation and MSE, althoughthe differences were small (mostly <5%). The markers in GBLUP are assumed to share the same normal distribution, whereas BayesC*π *fits only a small fraction of the available markers with an assumption that most loci are expected to have zero contribution to the independent variable, and the remaining nonzero marker effects are normally distributed. It is possible that the number of causal loci for SBP is relatively small, which is closer to the assumption of BayesC*π*. Similar improvement of BayesC*π *over GBLUP was found by previous studies [17,18].

**Table 1:** The accuracies of genomic prediction for additive genetic contribution to SBP.

SNPset	Model	TRN			TST	
	
		Within-family	Across-family		Within-family	Across-family
Set 1	GBLUP	0.844 (9.80)	0.823 (6.93)		0.348 (3.35)	0.062 (12.09)
Set 2	GBLUP	0.850 (9.70)	0.828 (6.85)		0.336 (3.39)	0.013 (12.16)
Set 1	BayesC*π*	0.883 (8.84)	0.854 (6.31)		0.351 (3.38)	0.054 (12.31)
Set 2	BayesC*π*	0.866 (9.45)	0.850 (6.50)		0.347 (3.36)	0.035 (12.11)

The accuracy is measured by correlation (MSE) between true and fitted additive genetic contributions from genomic predictions in the TRN and TST using SNP set 1 (common SNPs) and set 2 (common and collapsed SNPs).

Validation designs of prediction greatly affected the prediction accuracy. In both BayesC*π *and GBLUP, the within-family prediction had a strong advantage over across-family prediction, achieving a higher correlation between predicted value and true value, as well as a decreased MSE. For within-family prediction, TST was formed by the descendants of people in TRN, that is, closely related to each other. For the across-family prediction, individuals in TST and TRN were from different families, that is, unrelated to each other. Thus, the relatedness of individuals between TRN and TST strongly affected the performance of prediction models. This result was consistent with a genomic prediction study of human height [4] and several studies on the impact of genetic relationship information on genomic prediction in animal breeding [19,20].

The results from BayesC*π *in within-family prediction indicated that 14% of total variants (ie, 141,278 SNPs), on average, in TRN contributed when using SNPset 1, and 32% of the additive variance was explained by these SNPs. Thus the whole genome sequence variants detected a large proportion of the heritability (32%). The rest of the heritability might result from rare variants. We attempted to explore an approach in our models to include rare variants, but the addition of the collapsed SNPs did not improve the prediction accuracies (performance of SNPset 2 vs. set 1 in Table 1). Prediction accuracies using SNP set 2 were consistent among multiple runs of k-means methods with different starting points. It is possible that only three clusters were not enough to capture the genetic effects of the combinations of 100 rare SNPs, or that different window sizes should be considered rather than fixed at 100, or the relationships between the clusters is more complicated than what we modeled by the coding of the 3 clusters to 0, 1, and 2 under an additive genetic effect assumption. Different implementations of k-means method should be explored in future studies. Other clustering strategies to collapse rare variants could be attempted as well.

In within-family prediction, there were 289 individuals from TST with at least 1parent in TRN. Based on the results from the linear regression model (6), the prediction accuracy (the R^2^) using pedigree based information only is 0.455, higher than the 0.353 using whole genome markers only. Combining information from both sources, the prediction accuracy of 0.458 slightly outperformed either of the single source prediction. Including the parent average breeding value in genomic evaluations in animals is a common practice [21], which leads to a significantly greater reliability compared to using parent average breeding value only. An advantage of the inclusion is to obtain any genetic variance not captured by markers, for example, low-frequency quantitative trait loci.

Finally, the population size in this study may not be enough to obtain highly reliable variance component and additive genetic contribution estimates, which can bring extra noise into genomic predictions. It is also possible that SBP has limited additive genetic influences (ie, the low heritability estimate) and is not a good candidate for genomic prediction. With the limitations of the Genetic Analysis Workshop (GAW) data set (blood pressure was the only outcome) and GAW timeline, we did not have an opportunity to explore the impact of our model choice. Strategies that may improve the accuracy of genomic prediction might be (a) increasing the reference population size, (b) using a trait with a higher heritability, and (c) including information of relatives in the reference population.

## Conclusions

By using prediction models borrowed from animal breeding, GBLUP, and BayesC*π*, we showed that prediction of additive genetic contributions for a complex trait using whole genome sequencing data in humans is feasible. The prediction accuracy is strongly affected by the relatedness of individuals between TRN and TST. A large proportion of the additive variance can be explained through inclusions of whole genome sequence information in the model. The k-means method as implemented in our study for inclusion of rare variants did not improve the prediction. Different implementations of k-means or other methods for including rare variants in genomic prediction should be tested. Including both genomic and parent average information in the prediction model gave a slightly better accuracy than using either one of them alone.

## Competing interests

The authors declare that they have no competing interests.

## Authors' contributions

CY designed the study and wrote the manuscript. NL conducted the k-means method and computations. CY and NL were responsible for statistical analysis. KAW provided guidance in statistical methodologies. CDE coordinated data acquisition through GAW and provided feedback on conceptual development. KJM and KEL provided oversight and feedback from conception of design through manuscript submission. All authors read, revised, and approved the final manuscript.
